# XYL-1 and Olaparib Synergistically Inhibit the Growth of Pancreatic Cancer by Suppressing the SCD1/BRCA1 Signaling Pathway

**DOI:** 10.3390/molecules31162781

**Published:** 2026-08-10

**Authors:** Ye Yang, Lei Huang, Yaru Du, Qingyue Zhu, Li Dai, Bingjun Qian

**Affiliations:** 1 Department of Pharmacology and Medicinal Chemistry, Jiangsu Medical College, Yancheng 224005, China; 18361073522@163.com; 2 Department of Chinese Traditional Medicine, Nanjing University of Chinese Medicine, Nanjing 210023, China; 15851884176@163.com (Y.D.); z260928491@163.com (Q.Z.); 3 Department of Preventive Medicine, Jiangsu Medical College, Yancheng 224005, China; daili@jsmc.edu.cn

**Keywords:** PARP7, PARP1/2, HR, SCD1, BRCA-proficient, pancreatic cancer

## Abstract

PARP1/2 inhibitors have received FDA approval for pancreatic cancer harboring BRCA1/2 mutations and homologous recombination (HR) deficiency; however, their limited indications restrict their broader clinical application. Previous studies have demonstrated that PARP7, a member of the PARP family, enhances tumor sensitivity to PARP1/2 inhibition. However, the mechanisms underlying their synergistic effects in pancreatic cancer remain unclear. Herein, we found that combined inhibition of PARP1/2 and PARP7 using Olaparib and XYL-1 significantly inhibited the proliferation of SW1990 and CFPAC cells compared with either single agent. Furthermore, XYL-1 and Olaparib cooperatively caused DNA damage and induced cell apoptosis in SW1990 cells. Consistently, combined treatment with XYL-1 and Olaparib significantly suppressed SW1990 tumor growth compared with single-agent treatment in mouse xenograft models, accompanied by elevated levels of phosphorylated H2AX in tumor tissues. Notably, bioinformatic analyses and mechanistic studies identified SCD1 and BRCA1 as key mediators of the synergistic antitumor effects of XYL-1 and Olaparib. More importantly, the combination of XYL-1 and Olaparib synergistically downregulated the expression of SCD1 and BRCA1, thereby impairing the HR-mediated DNA repair pathway. Collectively, these findings suggest that dual targeting of PARP7 and PARP1/2 may represent a promising therapeutic strategy for BRCA-proficient pancreatic cancer.

## 1. Introduction

Pancreatic cancer is an exceptionally aggressive malignant disease, with its global incidence increasing by approximately 1.1% annually [1]. Epidemiological data predict that it will become the second leading cause of cancer-related deaths worldwide by 2030 [2]. Most patients are diagnosed at an advanced stage, leaving limited therapeutic options. Consequently, the overall five-year survival rate for pancreatic cancer remains extremely low, at approximately 11%. In recent years, the rapid advancement of molecular targeted therapy has opened new avenues for the treatment of this disease. Olaparib, a well-known PARP1/2 inhibitor, has demonstrated significant clinical benefits in the treatment of metastatic pancreatic cancer harboring BRCA1/2 mutations by targeting the DNA damage response (DDR) pathway [3,4].

Poly (ADP-ribose) polymerases (PARPs), particularly PARP1 and PARP2, play critical roles in the DDR pathway. PARP1/2 facilitate the repair of DNA single-strand breaks (SSBs) through the base excision repair (BER) pathway [5]. The unrepaired SSBs can be converted into lethal DNA double-strand breaks (DSBs) during the DDR process, ultimately leading to genomic instability and cell death. Homologous recombination (HR) is the most accurate mechanism for DSB repair, utilizing a sister chromatid as a template to ensure faithful DNA repair [6]. The tumor suppressor genes breast cancer susceptibility gene 1 and 2 (BRCA1 and BRCA2) are critical components of the HR pathway and play critical roles in maintaining genomic integrity through DSB repair [7,8]. Deficiency or mutation of BRCA1/2 impairs HR-mediated DNA repair, rendering tumor cells highly dependent on PARP1/2-mediated repair pathways for survival [9]. This vulnerability provides the theoretical basis for the synthetic lethality induced by PARP1/2 inhibitors, a therapeutic strategy that has demonstrated remarkable efficacy in cancers harboring BRCA1/2 mutations. Although PARP1/2 inhibitors have shown significant clinical benefits in patients with BRCA-mutated pancreatic cancer, the relatively low prevalence of BRCA mutations limits the broader application of these agents [10]. Therefore, identifying effective strategies to sensitize BRCA-proficient pancreatic tumors to PARP1/2 inhibition is of considerable clinical importance.

More studies have focused on developing combination strategies involving various new targets or reagents to overcome the limitations of PARP1/2 inhibitors [11,12,13]. It is becoming increasingly evident that the inhibition of enzyme activity to induce HR deficiency has emerged as a major strategy for expanding the clinical applicability of PARP1/2 inhibitors [14]. PARP7 (also known as ARTD14), a member of the PARP enzyme family, has been reported to be involved in cell proliferation, immunity, apoptosis, lipid metabolism, and other biological processes [15,16]. PARP7 is highly expressed in various cancers, including pancreatic cancer, and promotes tumor cell immune evasion by suppressing the type I interferon signaling pathway. Inhibition of PARP7 has been reported to increase tumor sensitivity to PARP1/2 inhibitors by activating the cGAS/STING signaling pathway, thereby strengthening the anti-tumor immune microenvironment [17]. Consequently, PARP7 has been recognized as a potential target for immunotherapy that can synergistically enhance antitumor effects. However, recent evidence suggests that PARP7 may also suppress tumor growth through mechanisms independent of immune regulation. For example, Spirtos AN et al. (2024) reported that RBN-2397, a selective PARP7 inhibitor, synergizes with paclitaxel to inhibit the proliferation and migration of ovarian cancer cells by regulating α-tubulin mono-ADP-ribosylation [18]. Furthermore, disruption of PARP7 induces a dense pan-tissue network of synthetic lethalities with chromatin remodeling factors and regulators of DNA repair [19]. Moreover, Thioparib, a dual inhibitor targeting PARP1/2 and PARP7, has been shown to inhibit HR-mediated DNA repair and circumvent PARP1/2 inhibitor resistance [20]. Recent studies have identified stearoyl-CoA desaturase 1 (SCD1), a key rate-limiting enzyme that catalyzes the conversion of saturated fatty acids into mono-unsaturated fatty acids, as an important regulator of cancer cell metabolism [21]. Beyond its established role in lipid metabolism, accumulating evidence indicates that SCD1 also contributes to the maintenance of genomic stability. Specifically, loss of SCD1 activity or accumulation of saturated fatty acids impairs HR-mediated DNA repair and induces apoptosis [22]. These findings suggest that SCD1 may serve as a possible molecular link between PARP7 and PARP1/2. Collectively, the findings gave rise to the hypothesis that the inhibition of PARP7 enzyme activity may render pancreatic cancer cells more sensitive to PARP1/2 inhibitors, through mechanisms that are independent of immune regulation.

In this study, the PARP7 inhibitor XYL-1 and the PARP1/2 inhibitor Olaparib were selected for investigation of their synergistic effect on the growth of BRCA-proficient pancreatic cancer cells and the underlying mechanisms, both in vitro and in vivo.

## 2. Results

### 2.1. Expression, Correlation, and Prognostic Analysis of PARP7 and PARP1/2 in Pancreatic Cancer Tissue

**Figure 1:**
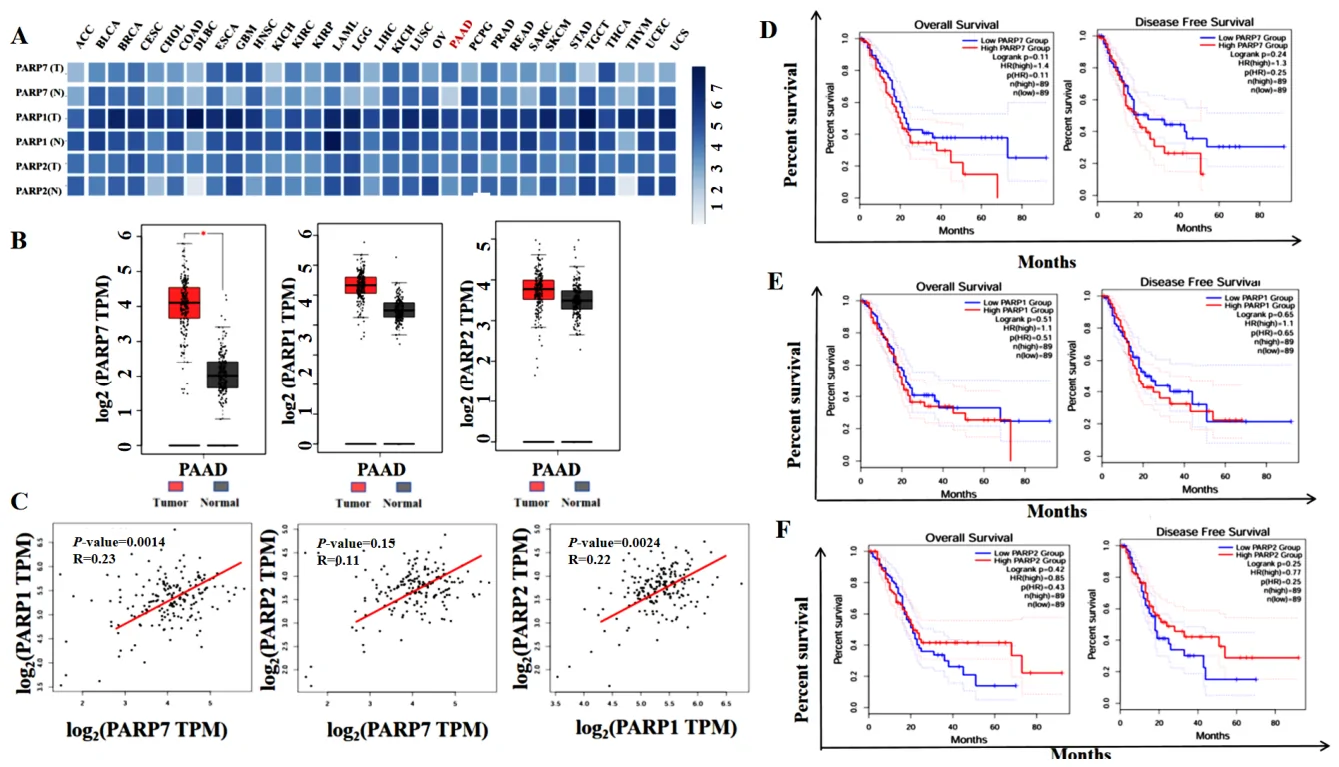
Expression, correlation, and prognostic analysis of PARP7, PARP1, and PARP2 using the online database GEPIA2 (http://gepia2.cancer-pku.cn accessed on 7 August 2026). (**A**) Differential expression levels of PARP1, PARP2, and PARP7 in tumor versus adjacent normal tissues across 31 cancer types from the TCGA database. (**B**) Differential expression levels of PARP1, PARP2, and PARP7 in pancreatic cancer tissues compared with adjacent normal tissues. (**C**) Correlation analysis of PARP1, PARP2, and PARP7 expression in pancreatic cancer tissues versus adjacent normal tissues. (**D**) Analysis of the association between patient survival and PARP7 expression. (**E**) Analysis of the association between patient survival and PARP1 expression. (**F**) Analysis of the association between patient survival and PARP2 expression. * *p* < 0.05 indicate significant differences compared with the normal group.

To assess the therapeutic potential of PARP7, an examination was first conducted of the expression levels of PARP7, PARP2, and PARP1 in tumor versus normal adjacent tissues across 31 cancer types from The Cancer Genome Atlas (TCGA) database. As demonstrated in Figure 1, the expression levels of PARP7 and PARP1 were significantly elevated in pancreatic cancer tissues compared with normal adjacent tissues (Figure 1A,B). Subsequently, an analysis was conducted of the correlations between PARP7, PARP1, and PARP2 expression using TCGA datasets. The results showed that PARP7 expression was positively correlated with PARP1 expression (R = 0.23, *p* < 0.01) and exhibited a weak positive correlation with PARP2 expression (R = 0.11, *p* = 0.15), which suggested that PARP7 and PARP1 could promote the expression of each other (Figure 1C). Finally, an investigation was conducted into the prognostic significance of PARP7, PARP1, and PARP2 expression in pancreatic cancer patients using TCGA data. The results showed that increased PARP7 expression was significantly associated with poorer overall survival (OS) and disease-free survival (DFS) in patients with pancreatic cancer (Figure 1D–F), indicating that increased PARP7 expression is associated with unfavorable prognosis. Collectively, these results suggest that PARP7 may serve as a promising therapeutic target for pancreatic adenocarcinoma (PAAD).

### 2.2. XYL-1 Plus Olaparib Exerts Synergistic Cytotoxicity in BRCA-Proficient Pancreatic Cancer Cell Lines

**Figure 2:**
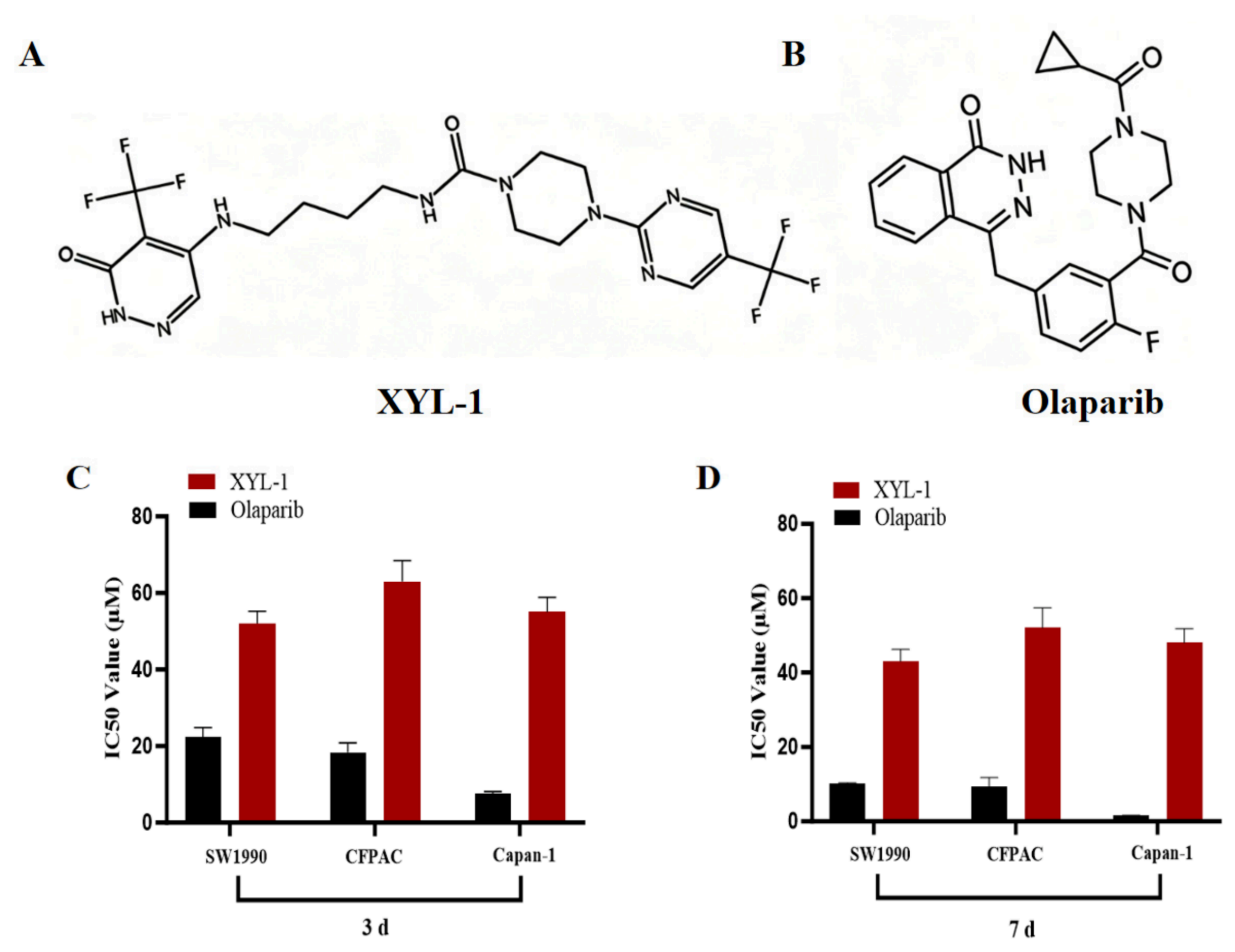

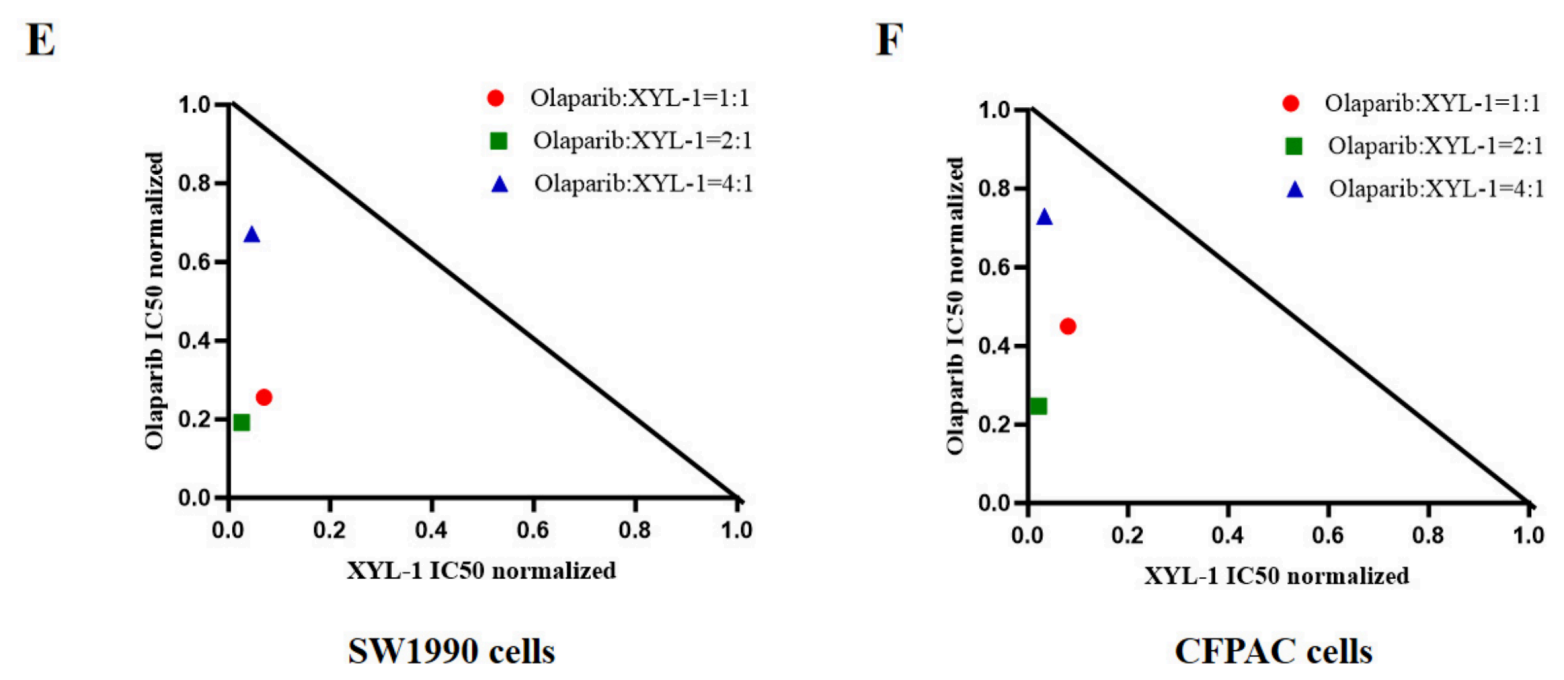
Synergistic effects of XYL-1 or/and Olaparib on the growth of pancreatic cancer cells. (**A**) The structure of the PARP7 inhibitor XYL-1. (**B**) The structure of the PARP1/2 inhibitor Olaparib. (**C**) Inhibitory activities of XYL-1 or Olaparib on SW1990, CFPAC, and Capan-1 for 3 days. (**D**) Inhibitory activities of XYL-1 or Olaparib on SW1990, CFPAC, and Capan-1 for 7 days. (**E**) Normalized IC_50_ isobologram showing synergistic effects of the XYL-1 and Olaparib combination for SW1990 cells. The combination ratios of XYL-1 and Olaparib were 1:1, 1:2, and 1:4. (**F**) Normalized IC_50_ isobologram showing synergistic effects of the Olaparib and XYL-1 combination for CFPAC cells. The combination ratios of XYL-1 and Olaparib were 1:1, 1:2, and 1:4. Values represent the mean from three separate experiments and error bars represent the standard error of the mean.

To further determine whether PARP7 and PARP1/2 inhibitors act synergistically, we evaluated the effects of XYL-1 and Olaparib on the viability of SW1990, CFPAC-1, and Capan-1 cells using the Cell Counting Kit-8 (CCK-8) assay (Figure 2A,B). Firstly, XYL-1 exhibited promising inhibitory activity against PARP7, with an IC_50_ value of 0.61 nM, confirming its high affinity for the PARP7 target (Table S1). As shown in Figure 2, XYL-1 alone did not significantly inhibit the proliferation of SW1990, CFPAC-1, and Capan-1 cells, which may be attributed to the absence of immune components in the in vitro culture system. However, Olaparib treatment inhibited the growth of SW1990, CFPAC-1, and Capan-1 cells in a time-dependent manner. After 7 days of treatment, the cytotoxic effect of Olaparib was the most pronounced across all three pancreatic cancer cell lines (Figure 2C,D). Notably, the inhibitory effect of Olaparib was markedly stronger in BRCA2-deficient Capan-1 cells in all pancreatic cancer cell lines, suggesting that BRCA status indeed influences sensitivity to PARP1/2 inhibitors (Figure 2C,D). We next evaluated the synergistic effects of XYL-1 and Olaparib using a fixed-ratio drug combination approach in SW1990 and CFPAC cells. Compared with either monotherapy, the combination of XYL-1 and Olaparib significantly inhibited the proliferation of SW1990 and CFPAC-1 cells at various molar ratios (Figure 2E,F). Combination index (CI) values were less than 1 in both cell lines, indicating a synergistic effect of the combination treatment (Table 1 and Table 2). Notably, the 1:2 ratio of XYL-1 to Olaparib produced the strongest synergistic cytotoxic effect in SW1990 cells. To further verify that this synergy resulted from PARP7 inhibition rather than off-target effects, we tested a second, structurally distinct PARP7 inhibitor, I-1. Similar to XYL-1, I-1 synergistically enhanced the antiproliferative activity of Olaparib in SW1990 cells, with the strongest effect again observed at a 1:2 molar ratio (Table S2). This combination did not significantly affect the viability of the normal human pancreatic epithelial cell line HPDE6-C7 (Table S3). Therefore, we selected the SW1990 cell line for subsequent experiments. Collectively, these findings demonstrate that XYL-1 and Olaparib exert synergistic cytotoxic effects in pancreatic cancer cells.

### 2.3. XYL-1 and Olaparib Synergistically Induces DNA Damage in SW1990 Cells

**Figure 3:**
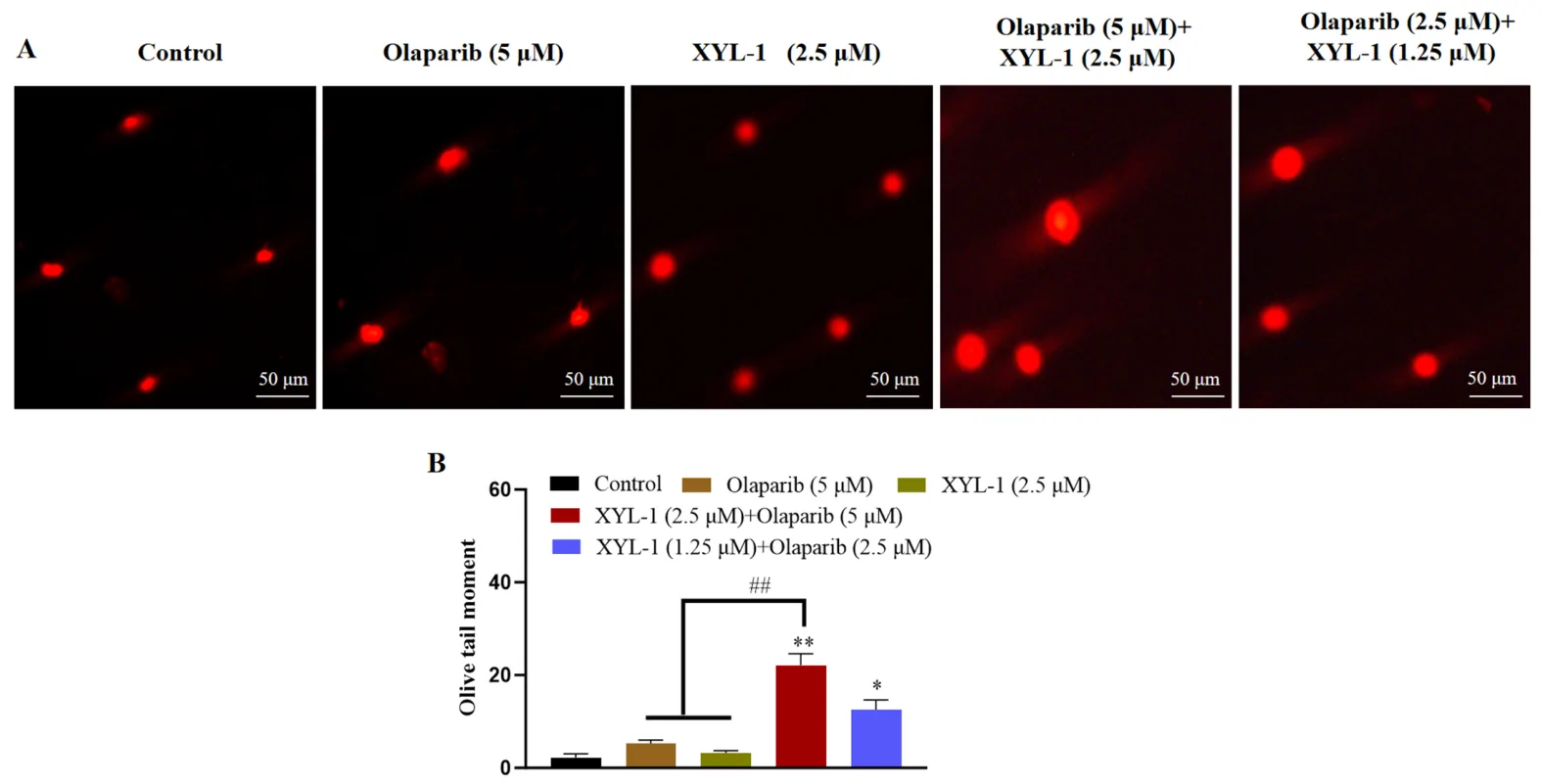
Effects of Olaparib or/and XYL-1 on DNA damage in SW1990 cells using the alkaline comet assay. (**A**) Representative images of the alkaline comet assay were taken by fluorescence microscope. All images were acquired under identical conditions (scale bars, 50 μm). (**B**) The results were analyzed quantitatively using the Comet Assay Software Project (CASP)version 1.2.2. The data are expressed as the mean ± standard deviation (SD) of three independent experiments (*n* = 3). Statistical significance was determined by one-way analysis of variance (ANOVA). * *p* < 0.05 and ** *p* < 0.01 indicate significant differences compared with the control group; ^##^ *p* < 0.01 indicates significant differences compared with the XYL-1/Olaparib combined group (2.5 + 5 μM).

To elucidate the reasons behind the synergistic cytotoxicity of XYL-1 combined with Olaparib on SW1990 cells, we employed the comet assay to assess the level of DNA damage in the cells. In this assay, damaged, fragmented DNA migrates toward the anode, producing a comet tail. As shown in Figure 3, after 7 days of treatment, XYL-1 alone produced almost no detectable comet tails, indicating a minimal effect on DNA damage. Similarly, the PARP1/2 inhibitor Olaparib did not significantly increase tail DNA levels in SW1990 cells. Compared with either XYL-1 or Olaparib monotherapy, the combination treatment increased both the number and extent of tailed DNA in a dose-dependent manner (Figure 3A,B). These results suggest that XYL-1 and Olaparib synergistically promote DNA damage in SW1990 cells.

### 2.4. XYL-1 and Olaparib Synergistically Promote Cell Apoptosis

**Figure 4:**
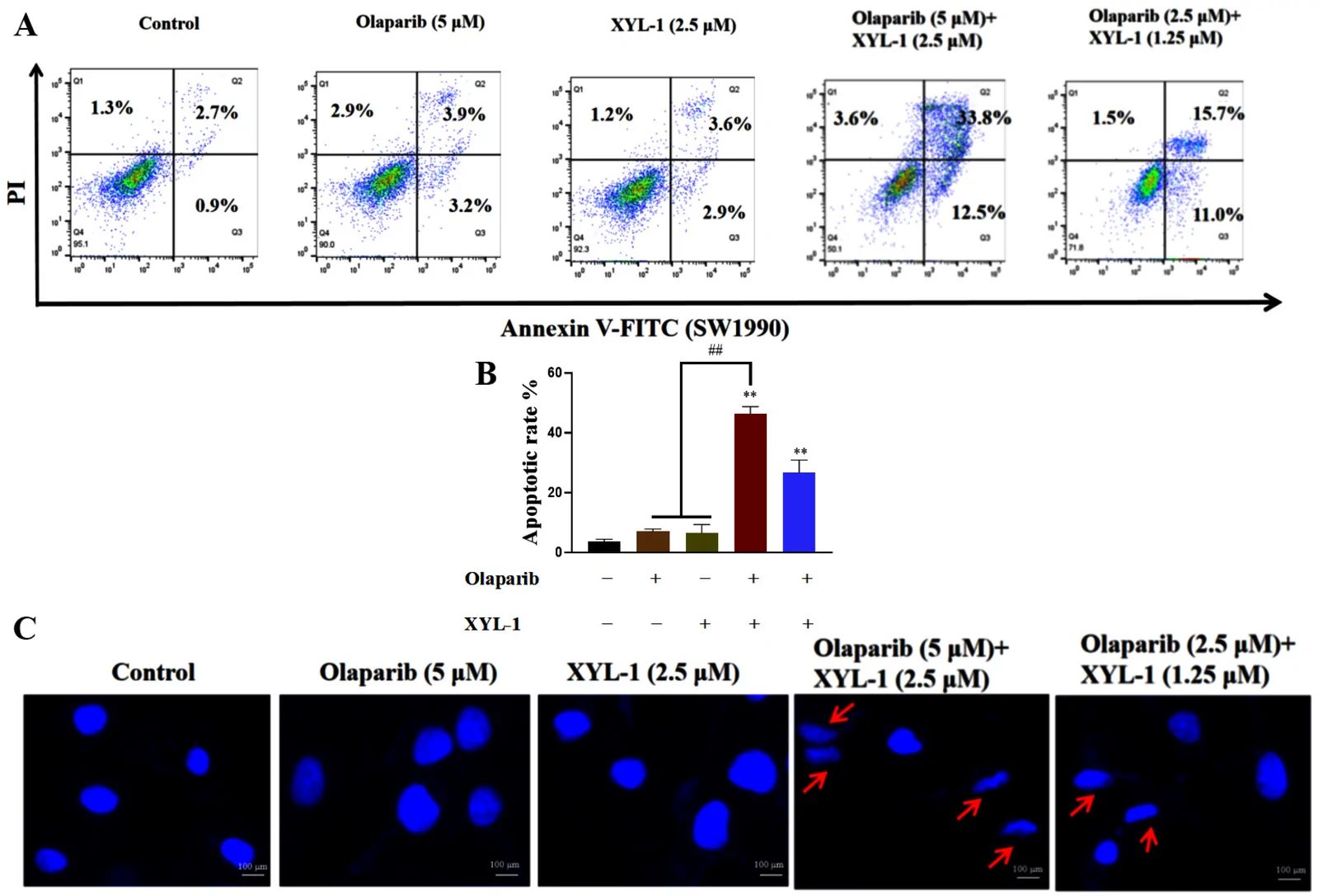
Effects of XYL-1 or/and Olaparib on apoptosis in SW1990 cells. (**A**) Apoptosis was analyzed by Annexin V-FITC/propidium iodide (PI) double staining followed by flow cytometry after 7 days of treatment with XYL-1 and/or Olaparib. Representative flow cytometry profiles are presented. (**B**) Quantitative analysis of apoptotic cells. (**C**) Apoptotic cells were further examined by Hoechst staining 33342. Representative confocal microscopy images are shown (cells exemplifying apoptotic nuclei are demarcated by red arrows). The data are expressed as the mean ± standard deviation (SD) of three independent experiments (*n* = 3). Statistical significance was determined by one-way analysis of variance (ANOVA). ** *p* < 0.01 indicate significant differences compared with the control group; ^##^ *p* < 0.01 indicates significant differences compared with the XYL-1/Olaparib combined group (2.5 + 5 μM). “+” means the compound was added and “−” means the compound was not added.

To evaluate the effects of XYL-1 and/or Olaparib on DNA damage-associated apoptosis in SW1990 cells, apoptosis was assessed by Annexin V-FITC/PI double staining followed by flow cytometric analysis. As illustrated in Figure 4A, treatment with either XYL-1 or Olaparib alone caused only a modest increase in apoptotic cells. In contrast, co-treatment with XYL-1 and Olaparib markedly elevated both the early and late apoptotic cell populations in a concentration-dependent manner compared with either single-agent treatment. In addition, apoptotic morphological changes were further evaluated by Hoechst 33258 staining. As shown in Figure 4B, compared with either monotherapy, the combination of XYL-1 and Olaparib exhibited significant nuclear pyknosis, chromatin condensation, and fragmentation, indicating a synergistic pro-apoptotic effect of the two drugs.

### 2.5. Olaparib and XYL-1 Synergistically Induce DNA Damage and Apoptosis Associated with Suppression of SCD1/BRCA1 Signaling Pathway

**Figure 5:**
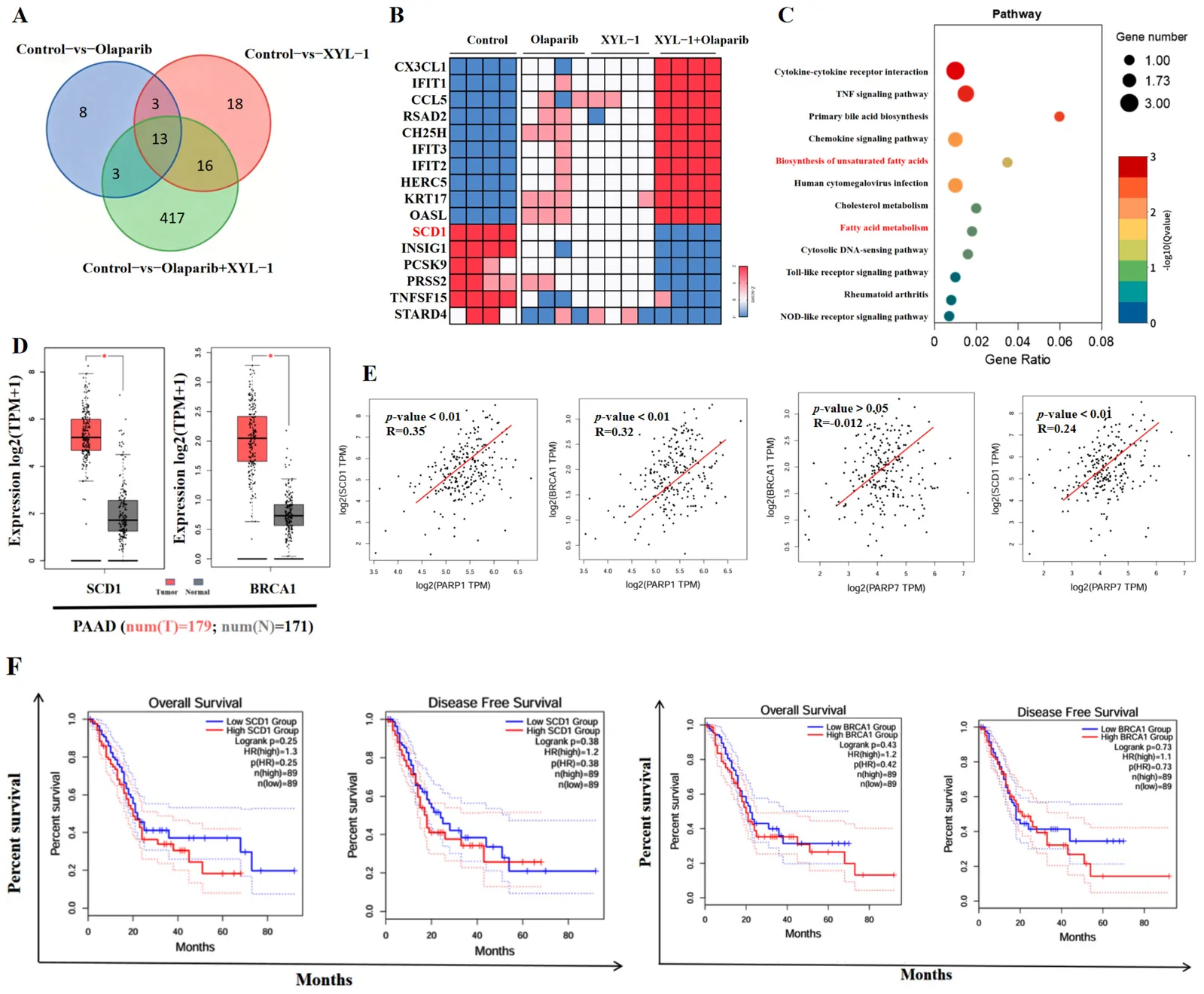

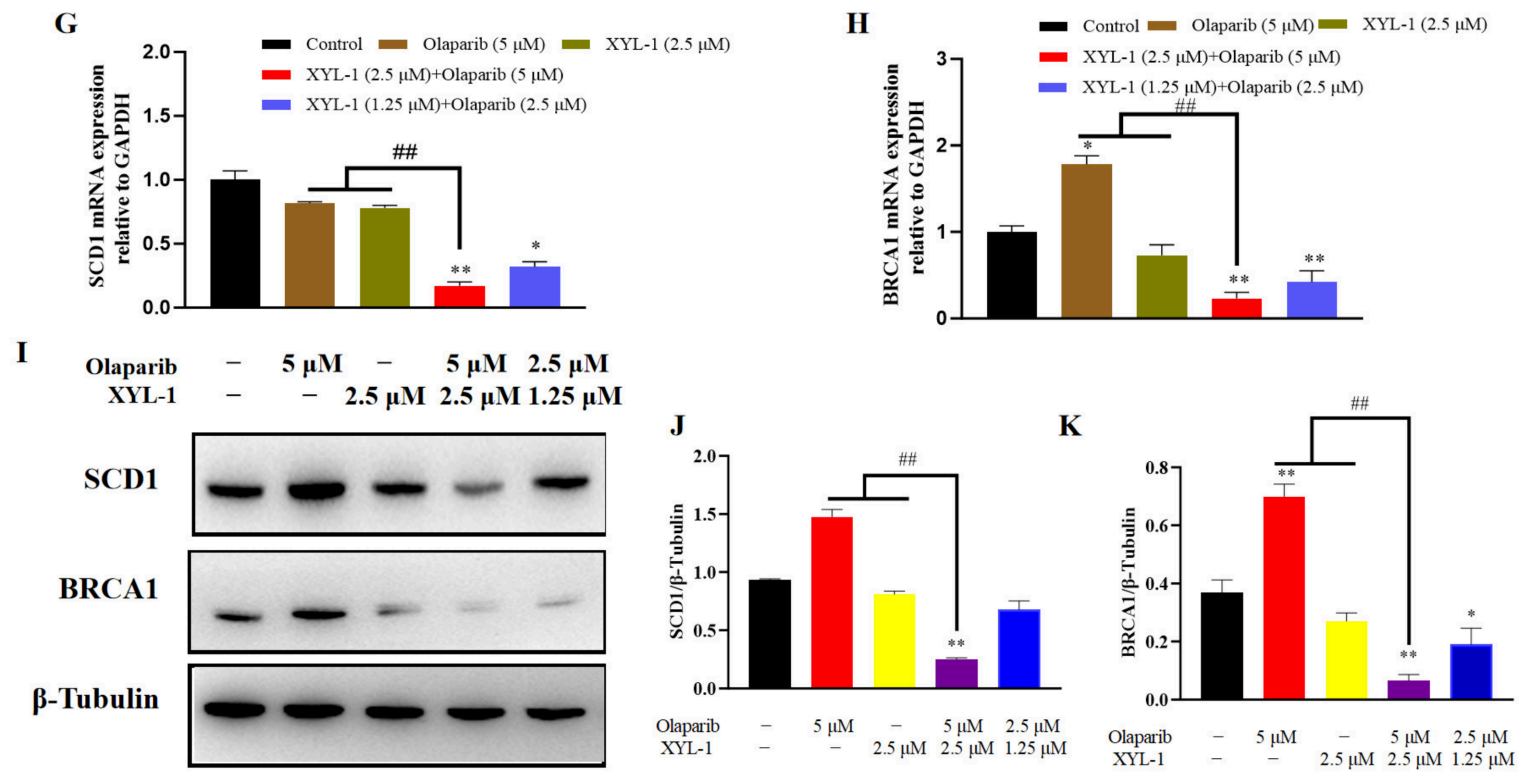
XYL-1 and Olaparib synergistically inhibit the expression of SCD1 and BRCA1. (**A**) Venn diagram illustrating the overlap of differentially expressed genes (DEGs) among the Olaparib, XYL-1, and combination treatment groups relative to the control group. (**B**) Heatmap of the common DEGs induced by the indicated treatments. (**C**) KEGG pathway enrichment analysis of the six common DEGs. (**D**) Differential expression analysis of SCD1 and BRCA1 in pancreatic cancer tissues and adjacent normal tissues based on TCGA datasets. * *p* < 0.05 indicate significant differences compared with the normal group. (**E**) Correlation analysis between SCD1 and BRCA1 expression in pancreatic cancer tissues. (**F**) Kaplan–Meier survival analysis according to SCD1 or BRCA1 expression levels in patients with pancreatic cancer. (**G**) Relative mRNA expression levels of SCD1 in SW1990 cells following treatment with XYL-1 and/or Olaparib, as determined by qRT-PCR. (**H**) Relative mRNA expression levels of BRCA1 in SW1990 cells following treatment with XYL-1 and/or Olaparib, as determined by qRT-PCR. (**I**) Representative Western blot images showing SCD1 and BRCA1 protein expression in SW1990 cells after treatment with XYL-1 and/or Olaparib. (**J**) Densitometric quantification of SCD1 protein expression. Band intensities were analyzed using ImageJ software and normalized to β-tubulin. (**K**) Densitometric quantification of BRCA1 protein expression. Band intensities were analyzed using ImageJ software and normalized to β-tubulin. The data are expressed as the mean ± standard deviation (SD) of three independent experiments (*n* = 3). Statistical significance was determined by one-way analysis of variance (ANOVA). * *p* < 0.05 and ** *p* < 0.01 indicate significant differences compared with the control group; ^##^ *p* < 0.01 indicates significant differences compared with the XYL-1/Olaparib combined group (2.5 + 5 μM). “+” means the compound was added and “−” means the compound was not added.

To investigate the molecular mechanisms underlying the synergistic effects of PARP1/2 and PARP7 inhibitors, we performed RNA sequencing (RNA-Seq) on SW1990 cells treated with Olaparib, XYL-1, or their combination. As shown in Figure 5, among these 16 common differentially expressed genes (DEGs), SCD1, INSIG1, PCSK9, PRSS2, and TNFSF15 were significantly downregulated after combination treatment with XYL-1 and Olaparib (Figure 5A,B). KEGG pathway enrichment analysis showed that these DEGs were predominantly enriched in pathways related to immune regulation, lipid metabolism, and signal transduction (Figure 5C). Given that SCD1 is critical for lipid metabolism and BRCA1 functions as a tumor suppressor in HR-mediated DNA repair, we selected these two genes for further bioinformatic analysis. As shown in Figure 5D, both SCD1 and BRCA1 were expressed at higher levels in pancreatic cancer tissues than in matched adjacent normal tissues. Correlation analysis further demonstrated positive associations of SCD1 and BRCA1 expression with PARP7 and PARP1 expression (Figure 5E). Meanwhile, SCD1 expression was significantly associated with the survival of patients with pancreatic cancer, and higher SCD1 expression was associated with poorer prognosis (Figure 5F).

The expression of SCD1 and BRCA1 in SW1990 cells was subsequently examined at both the mRNA and protein levels by qRT-PCR and Western blotting. As shown in Figure 5G,H, Olaparib treatment for 7 days significantly increased BRCA1 mRNA expression, consistent with a compensatory activation of HR-mediated DNA repair. In contrast, co-treatment with XYL-1 and Olaparib markedly decreased the mRNA expression of both SCD1 and BRCA1, thereby abolishing the Olaparib-induced upregulation of BRCA1. Consistent with the mRNA expression results, Western blot analysis demonstrated that the protein expression levels of SCD1 and BRCA1 were significantly reduced following combination treatment compared with either monotherapy (Figure 5I,J). More importantly, similar effects were also observed with another structurally distinct, PARP7 inhibitor I-1 (Figure S6). In addition, RAD51 foci formation, a well-established functional marker of HR repair, was markedly reduced after the combination treatment, further supporting the impairment of HR-mediated DNA repair (Figure S2). To further investigate the functional role of SCD1 and BRCA1, we established stable SCD1-knockdown SW1990 cells using a lentivirus-mediated shRNA approach. Knockdown of SCD1 in SW1990 cells significantly inhibited cell proliferation and markedly reduced BRCA1 expression (Figure S3). Additionally, SCD1 knockout further decreased RAD51 foci formation, indicating that SCD1 is involved in HR repair (Figure S4). Overexpression of BRCA1 in SCD1-knockout cells promotes cell proliferation and RAD51 foci formation, suggesting that SCD1 acts, at least in part, through BRCA1 (Figures S5 and S6). Together, these findings suggest that the combination treatment suppresses SCD1/BRCA1 signaling pathway, which may be associated with impaired HR-mediated DNA repair.

### 2.6. XYL-1 and Olaparib Synergistically Suppressed the Growth of SW1990-Derived Xenograft Models

**Figure 6:**
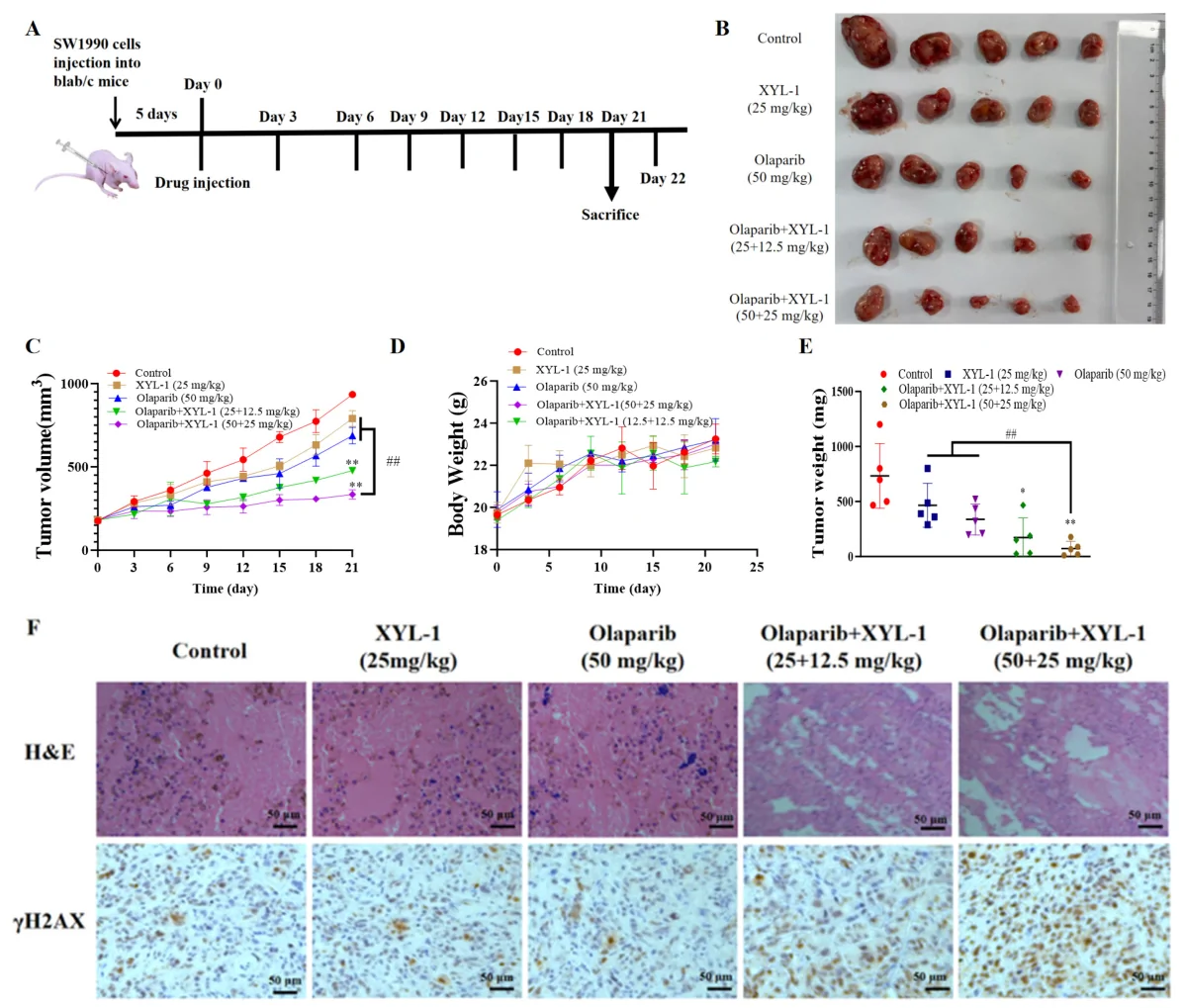
Antitumor efficacy of XYL-1 combined with Olaparib in the SW1990 xenograft model. (**A**) Experimental workflow for the establishment and treatment of the SW1990 xenograft mouse model. (**B**) Representative images of tumors excised from mice treated with vehicle, Olaparib, XYL-1, or the combination at the end of the study. (**C**) Changes in tumor volume during the treatment period. (**D**) Body weight changes in mice throughout the treatment period. (**E**) Final tumor weights measured at the experimental endpoint. (**F**) Representative hematoxylin and eosin (H&E) and immunohistochemical (IHC) staining of xenograft tumor sections. Data are presented as mean + SD, *n* = 5 mice per group from a single experiment.. Statistical significance was determined by one-way analysis of variance (one-way ANOVA). * *p* < 0.05 and ** *p* < 0.01 compared with the control group, ^##^ *p* < 0.01 compared with the combination group of XYL-1 and Olaparib (25 + 50 mg/kg).

To investigate the antitumor efficacy of XYL-1 in combination with Olaparib in vivo, a subcutaneous SW1990 xenograft model was established in nude mice. Once tumors reached an average volume of approximately 90–100 mm^3^, the mice were randomly assigned to five treatment groups: 0.9% normal saline (NS), Olaparib (50 mg/kg), XYL-1 (25 mg/kg), Olaparib plus XYL-1 (25 + 12.5 mg/kg), and Olaparib plus XYL-1 (50 + 25 mg/kg) (Figure 6). After 21 days of continuous treatment, either Olaparib or XYL-1 alone produced a mild inhibitory effect on tumor growth. In contrast, combined treatment with Olaparib and XYL-1 significantly suppressed SW1990 tumor growth in a dose-dependent manner (Figure 6A–E). More importantly, no significant differences in body weight were observed among the experimental groups throughout the treatment period, suggesting that the combination regimen was well-tolerated. In addition, hematoxylin and eosin (H&E) staining revealed distinct histopathological alterations in tumor tissues from mice receiving the combination treatment (Figure 6F). Immunohistochemical results further showed that the expression of phosphorylated H2AX, a biomarker of DNA damage, was substantially upregulated in the tumor tissues of mice receiving the combined therapy (Figure 6F). These findings indicate that the combination of Olaparib and XYL-1 effectively inhibits tumor growth and enhances DNA damage in vivo, thereby contributing to tumor suppression.

### 2.7. XYL-1 and Olaparib Synergistically Inhibit Tumor Growth in SW1990 Tumor Xenografts by Suppressing the SCD1 and BRCA1 Expression

**Figure 7:**
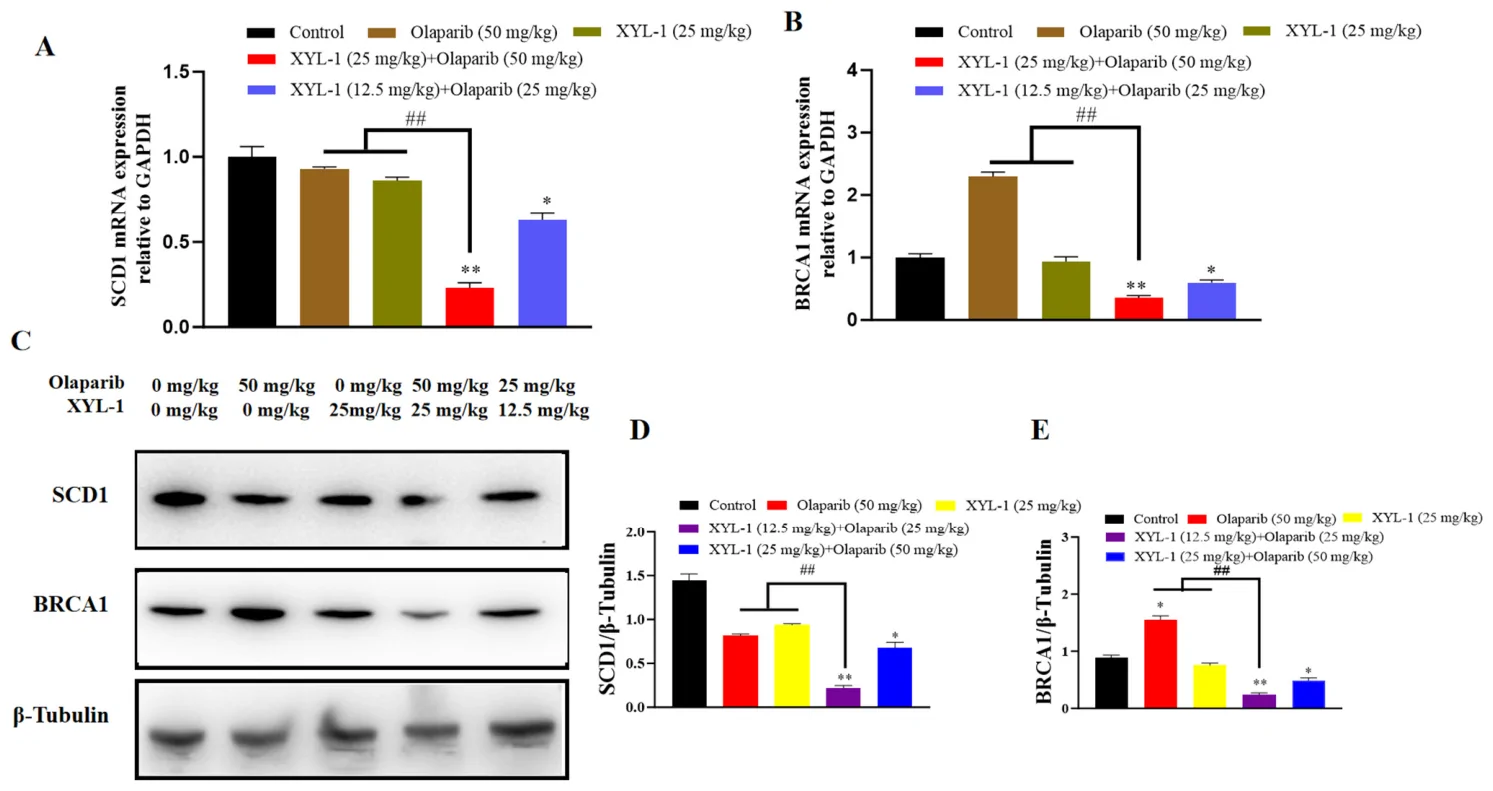
Effects of XYL-1 and/or Olaparib on the expression of SCD1 and BRCA1 in the SW1990 xenograft model. (**A**) The expression of SCD1 mRNA in the SW1990 xenograft model treated with XYL-1 and/or Olaparib was detected using qRT-PCR. (**B**) The expression of BRCA1 mRNA in the SW1990 xenograft model treated with XYL-1 and/or Olaparib was detected using qRT-PCR. (**C**) The expression levels of SCD1 and BRCA1 proteins in the SW1990 xenograft model treated with XYL-1 and/or Olaparib were detected using Western blotting. (**D**) The gray values of the SCD1 protein bands were quantitatively analyzed using ImageJ software, normalized to β-tubulin as a reference. (**E**) The gray values of the BRCA1 protein bands were quantitatively analyzed using ImageJ software, normalized to β-tubulin as a reference. Data are presented as mean + SD, *n* = 5 mice per group from a single experiment. Statistical significance was determined by one-way analysis of variance (one-way ANOVA). * *p* < 0.05 and ** *p* < 0.01 compared with the control group, ^##^ *p* < 0.01 compared with the combination group of XYL-1 and Olaparib.

To investigate whether PARP7 inhibition enhances the antitumor efficacy of PARP1/2 inhibition in vivo and whether the combination treatment affects SCD1 and BRCA1 expression, we established SW1990 xenograft models and evaluated the effects of XYL-1, Olaparib, and their combination. qRT-PCR analysis was performed to determine the mRNA expression levels of SCD1 and BRCA1 in tumor tissues. As shown in Figure 7A, treatment with Olaparib significantly increased BRCA1 mRNA expression, suggesting a compensatory activation of the HR-mediated DNA repair response. However, co-treatment with XYL-1 and Olaparib significantly reduced the mRNA expression levels of both SCD1 and BRCA1 compared with either monotherapy. Furthermore, Western blot analysis further confirmed that combined treatment markedly reduced the protein expression of SCD1 and BRCA1. This inhibitory effect was more pronounced than that observed with either XYL-1 or Olaparib alone and was consistent with the qRT-PCR results (Figure 7B–D). Taken together, these data indicate that the combination of XYL-1 and Olaparib effectively inhibits tumor growth in vivo while concomitantly decreasing SCD1 and BRCA1 expression, suggesting that modulation of the SCD1/BRCA1 axis may contribute to the antitumor activity elicited by the dual inhibition of PARP7 and PARP1/2.

## 3. Discussion

In this study, we demonstrated that the combination of the PARP1/2 inhibitor Olaparib and the PARP7 inhibitor XYL-1 exerted synergistic cytotoxic effects in SW1990 models, as evidenced both in vitro and in vivo. In vitro studies confirmed that Olaparib and XYL-1 synergistically inhibited cell proliferation, enhanced DNA damage, and promoted apoptosis. Further mechanistic studies revealed that XYL-1 and Olaparib significantly suppressed SCD1 and BRCA1 expression, thereby impairing HR-mediated DNA repair. Consistently, subsequent in vivo studies demonstrated that Olaparib and XYL-1 produced synergistic antitumor activity in xenograft-bearing mice while maintaining a favorable safety profile.

Targeting PARP1/2 has emerged as a promising therapeutic strategy for pancreatic cancer. However, the efficacy of PARP1/2 inhibitor monotherapy remains limited in BRCA-proficient tumors. A phase III clinical trial reported an objective response rate (ORR) of 59.9% and a median progression-free survival (PFS) of 7.0 months with Olaparib in pancreatic cancer patients harboring BRCA1/2 mutations [23]. In contrast, patients without BRCA1/2 mutations or other HR deficiencies derive minimal benefit from PARP1/2 inhibitor monotherapy, underscoring the need for novel combination strategies to suppress the progression of BRCA-proficient pancreatic cancer. Previous studies have indicated that combining PARP1/2 inhibitors with other agents may produce complementary cytotoxic effects [24,25]. In this study, we found that the combination of Olaparib and XYL-1 synergistically inhibited the growth of SW1990, CFPAC, and Capan-1 cells, with a 1:2 molar ratio of XYL-1 to Olaparib producing the strongest synergy in SW1990 cells. The cytotoxicity of PARP1/2 inhibition primarily arises from defective DNA repair and subsequent genomic instability. PARP7 disruption has also been shown to promote DNA damage in tumor cells and increase genomic instability [19]. Using comet assays and Hoechst staining experiments, we found that XYL-1 markedly enhanced Olaparib-induced DNA damage and promoted apoptosis, consistent with these earlier observations.

A previous study demonstrated that Thioparib, a dual PARP1/2 and PARP7 inhibitor, overcame Olaparib resistance by activating the type I interferon response [20]. Building on these findings, we investigated whether additional molecular mechanisms contribute to the synergistic antitumor activity of Olaparib and XYL-1. Our results showed that combined treatment with Olaparib and XYL-1 significantly increased phosphorylated H2AX accumulation in SW1990 xenograft nude mice, indicating impaired DNA DSB repair. γH2AX is rapidly recruited to DNA damage sites, where it facilitates the recruitment of repair factors and activates DNA damage response signaling, particularly during HR-mediated DSB repair [26]. In addition, BRCA1 and BRCA2 are key components of the HR pathway and localize to damaged DNA sites to participate in DSB repair [27]. We therefore examined BRCA1 and BRCA2 expression and found that XYL-1 and Olaparib reduced BRCA1 and γH2AX levels, whereas the combination did not further affect BRCA2 expression. Park et al. reported that BRCA1 is involved in DNA end resection and RAD51 loading, whereas BRCA2 is essential for RAD51 loading onto resected single-stranded DNA [28]. We therefore speculate that XYL-1 and Olaparib mainly affect the early stages of HR repair.

Multiple mechanisms may contribute to HR impairment, including metabolic reprogramming, immunomodulation, and dysregulated DNA damage responses. Based on RNA sequencing analysis, lipid metabolism-related pathways were significantly enriched following combined treatment with Olaparib and XYL-1. Among these pathways, SCD1 was notably downregulated in the combination group. SCD1 is a key lipid-modifying enzyme that catalyzes the desaturation of saturated fatty acids and is frequently overexpressed in a variety of malignancies, including pancreatic cancer [29]. Previous studies have shown that ovaries from SCD1^−/−^ mice exhibit dysregulated expression of RAD51, accompanied by increased DNA damage, indicating that SCD1 deficiency disrupts HR-associated signaling pathways [30]. Moreover, reduced SCD1 activity has been reported to trigger PARP1 hyperactivation followed by its degradation, thereby compromising HR repair capacity and promoting cell death [31]. Thus, SCD1 may protect various cancer cells from DNA damage caused by impaired HR, including pancreatic cancer cells. Collectively, our findings indicate that combined treatment with Olaparib and XYL-1 is associated with the downregulation of SCD1 and BRCA1 expression, which may contribute to impaired HR-mediated DNA repair and enhanced DNA damage accumulation in BRCA-proficient pancreatic cancer cells.

It is imperative to acknowledge the limitations of this study. Initially, only Olaparib and XYL-1 were evaluated; it remains to be determined whether the observed synergy effect extends to other PARP1/2 and PARP7 inhibitors. Secondly, SCD1 has been reported to regulate ferroptosis, thereby contributing to tumor resistance [32]. It is imperative that further investigation is conducted to ascertain whether this contributes to the impairment of HR repair induced by Olaparib and XYL-1. Finally, additional HR repair factors and immune-related mechanisms were not systematically examined, and further studies are warranted.

In summary, the present study demonstrated that the combination of Olaparib and XYL-1 exerted synergistic cytotoxic effects against SW1990 pancreatic cancer cells. Mechanistically, the combination of Olaparib and XYL-1 resulted in the promotion of cell apoptosis by enhancing the accumulation of DNA damage. This process may be, at least in part, mediated through the downregulation of SCD1 and BRCA1 expression. The findings presented herein provide preclinical evidence to support the hypothesis that the combined use of PARP1/2 and PARP7 inhibitors is a promising therapeutic strategy for patients with pancreatic cancer.

## 4. Materials and Methods

### 4.1. Reagents and Antibodies

The following primary antibodies, including BRCA1 (rabbit polyclonal, 1:1000, #9010), SCD1 (rabbit monoclonal, 1:1000, #2794), phospho-histone H2A.X (Ser139) (γH2AX; rabbit monoclonal, 1:1000, #9718), and β-tubulin (rabbit monoclonal, 1:1000, #2128), were purchased from Cell Signaling Technology (Danvers, MA, USA). PARP7 inhibitor XYL-1 and PARP1/2 inhibitor Olaparib were obtained from Selleck Chemicals (Houston, TX, USA). RPMI-1640 medium, Iscove’s modified Dulbecco’s medium (IMDM), Leibovitz’s L-15 medium, and bovine serum albumin (BSA) were purchased from Sigma-Aldrich (St. Louis, MO, USA). The Annexin V-FITC Apoptosis Detection Kit, Hoechst 33342 Staining Kit, Cell Counting Kit-8 (CCK-8), and Comet Assay Kit were supplied by KeyGEN Biotech (Nanjing, China).

### 4.2. Cell Culture

The pancreatic cancer cell lines used in this study were obtained from the Cell Resources Center of Shanghai Academy of Life Sciences (Shanghai, China). All cell lines were authenticated using short tandem repeat (STR) profiling and confirmed to match the respective ATCC reference strains: SW1990 (ATCC^®^ CRL-2172™), CFPAC-1 (ATCC^®^ CRL-1918™), and Capan-1 (ATCC^®^ HTB-79™, BRCA2-deficiency). Regular mycoplasma testing was performed throughout the cell culture process, and all cells were confirmed to be mycoplasma-free. SW1990 cells were cultured in Leibovitz’s L-15 medium supplemented with 10% (*v*/*v*) fetal bovine serum (FBS; Grand Island, NY, USA) and 1% (*v*/*v*) penicillin–streptomycin. CFPAC-1 cells were maintained in RPMI-1640 medium with the same supplementation of 10% (*v*/*v*) FBS and 1% (*v*/*v*) penicillin–streptomycin. Capan-1 cells were cultured in Iscove’s modified Dulbecco’s medium (IMDM), also supplemented with 10% (*v*/*v*) FBS and 1% (*v*/*v*) penicillin–streptomycin. All cultures were kept in a humidified incubator at 37 °C in a 5% CO_2_ atmosphere and were passaged regularly every 3–4 days. The cells were inoculated at an appropriate density to prevent them from reaching complete confluence before the end of the experiment.

### 4.3. Drug Combination Assays

Briefly, cells were plated in 96-well plates at a density of 1 × 10^3^ cells/well and incubated for 24 h at 37 °C under humidified conditions with 5% CO_2_. Subsequently, cells were exposed to different concentrations of XYL-1 and Olaparib, with 0.5% DMSO serving as the vehicle control. For drug combination experiments, XYL-1 and Olaparib were combined at fixed molar ratios of 1:1, 1:2, or 1:4. SW1990, CFPAC-1, and Capan-1 cells were continuously treated with the indicated drug combinations for 7 days, after which cell viability was evaluated using the CCK-8 assay. Drug interactions were analyzed by calculating combination index (CI) values using CompuSyn software version 1.0.1 (ComboSyn Inc., Paramus, NJ, USA) according to the Chou–Talalay method [33]. The CI was determined using the equation: CI = CA, X/ICX, A + CB, X/ICX, B, where CA, X and CB, X indicate the concentrations of XYL-1 and Olaparib, respectively, in the combination treatment required to achieve a given effect level (50% growth inhibition), and ICX, A and ICX, B represent the concentrations of the individual drugs required to generate the same effect. CI values < 1.0 indicate synergism, CI = 1.0 indicates an additive effect, and CI > 1.0 indicates antagonism.

### 4.4. Comet Assay

DNA damage was assessed by the alkaline comet assay according to a previously reported protocol with minor modifications [34]. Briefly, SW1990 cells were cultured in 6-well plates for 24 h to allow attachment and then exposed to XYL-1 and/or Olaparib for 7 days under standard culture conditions (37 °C, 5% CO_2_). Following drug treatment, cells were collected and adjusted to a concentration of 2 × 10^4^ cells/mL. Cell suspensions were combined with low-melting-point agarose at a 1:10 (*v*/*v*) ratio and rapidly transferred onto comet assay slides. After the agarose layer was solidified, slides were subjected to lysis in buffer at 4 °C for 2 h, followed by DNA unwinding in alkaline solution for 30 min at room temperature. Electrophoresis was performed at 21 V for 30 min under alkaline conditions. Subsequently, slides were stained with propidium iodide (PI), and DNA damage was visualized using an Olympus BX53 fluorescence microscope (Olympus, Tokyo, Japan). Image acquisition and quantitative analysis were conducted by investigators blinded to the treatment groups. DNA damage was assessed by analyzing comet tail formation. The quantitative analysis of comet images was performed using the Comet Assay Software Project (CASP), with DNA damage expressed as the percentage of tail DNA and the Olive tail moment (OTM). For quantitative analysis, at least 100 intact, non-overlapping comets were randomly selected from multiple microscopic fields for each independent biological replicate, and three independent experiments were conducted.

### 4.5. Analysis of Apoptosis

Apoptosis was quantified using an Annexin V-FITC/Propidium Iodide (PI) Apoptosis Detection Kit (KeyGEN Biotech, Nanjing, China) according to the manufacturer’s protocol. Briefly, cells were seeded in 8-well plates at a density of 2 × 10^5^ cells per well and cultured overnight before exposure to XYL-1 and/or Olaparib for 7 days. After treatment, cells were harvested, washed twice with ice-cold phosphate-buffered saline (PBS), and then incubated with Annexin V-FITC and PI for 15 min at room temperature in the dark. Stained cells were analyzed using a FACSCelesta flow cytometer (BD Biosciences, San Jose, CA, USA). Flow cytometry data were analyzed with FlowJo software (version 7.6; Tree Star Inc., Ashland, OR, USA). The percentage of total apoptotic cells was determined by combining the proportions of cells in early and late apoptotic stages.

### 4.6. Hoechst Staining

Nuclear morphological changes associated with apoptosis were assessed using a Hoechst 33258 staining kit (Beyotime Biotechnology, Shanghai, China) according to the manufacturer’s instructions. Briefly, cells were seeded in 6-well plates and incubated for 24 h before treatment with different concentrations of Olaparib and/or XYL-1 for 7 days. Cells without drug exposure served as the control group. Following treatment, cells were washed with PBS and fixed with 4% paraformaldehyde for 15 min at room temperature. After fixation, cells were washed twice with PBS and incubated with Hoechst 33258 staining solution for 5 min. The cells were then washed twice with PBS, and nuclear morphology was visualized using a fluorescence microscope (Zeiss, Oberkochen, Germany).

### 4.7. Western Blot Assay

Total proteins were isolated from xenograft tumor tissues and SW1990 cells using RIPA lysis buffer (Beyotime Biotechnology, Shanghai, China) supplemented with phosphatase inhibitor cocktails and protease. After lysis, samples were centrifuged at 12,000× *g* for 15 min at 4 °C, and the resulting supernatants were collected for protein quantification using a BCA protein assay kit (Beyotime Biotechnology, Shanghai, China). Equal amounts of protein were resolved by SDS-PAGE and electrotransferred onto PVDF membranes (Merck Millipore, Billerica, MA, USA). The membranes were blocked with 5% nonfat milk (*w*/*v*) for 2 h at room temperature and then incubated with the appropriate primary antibodies overnight at 4 °C. After washing, the membranes were incubated with HRP-conjugated secondary antibodies (1:5000) for 2 h at room temperature. Immunoreactive bands were detected using an enhanced chemiluminescence (ECL) system and quantified by densitometric analysis with ImageJ software 1.8.0 version (National Institutes of Health, Bethesda, MD, USA). β-Tubulin was used as the loading control.

### 4.8. Pharmacodynamics Studies in the SW1990 Xenograft Model

Female NOD/SCID/IL2Rγ-null (NSG) mice (6–8 weeks old, 18–22 g) were obtained from Qinglongshan Animal Breeding Center (Nanjing, China). Animals were housed in a specific pathogen-free (SPF) environment with unrestricted access to food and water. All experimental procedures involving animals were reviewed and approved by the Institutional Animal Care and Use Committee of Jiangsu Medical College (Yancheng, China) and conducted in accordance with the Guide for the Care and Use of Laboratory Animals. For tumor xenograft generation, SW1990 cells (5 × 10^6^ cells in PBS) were injected subcutaneously into the right flank of each mouse. After tumors had formed and reached approximately 100 mm^3^, mice were randomly divided into five groups (*n* = 5/group): vehicle control (0.9% NS), XYL-1 (25 mg/kg), Olaparib (50 mg/kg), XYL-1 (25 mg/kg) combined with Olaparib (50 mg/kg), and XYL-1 (12.5 mg/kg) combined with Olaparib (25 mg/kg). Mice received intraperitoneal administration of the indicated treatments once daily for 21 consecutive days, while control animals were injected with an equivalent volume of normal saline. Tumor dimensions and body weights were recorded every two days during the treatment period. Tumor volume was calculated using the formula: volume (mm^3^) = 0.5 × length × width^2^. At the end of the study, mice were euthanized by cervical dislocation, and xenograft tumors were harvested for further analysis. Throughout the experiment, all procedures were performed with the aim of minimizing animal distress.

### 4.9. Immunohistochemistry

At the completion of the study, mice were euthanized, and xenograft tumors were collected and immediately fixed with 4% paraformaldehyde. After fixation, tumor samples were embedded in paraffin and cut into 4-μm-thick sections. The tissue sections were deparaffinized with xylene, rehydrated through a graded series of ethanol solutions, and subjected to heat-induced antigen retrieval in citrate buffer. To eliminate endogenous peroxidase activity, sections were incubated with 3% hydrogen peroxide for 15 min at room temperature. After rinsing with phosphate-buffered saline (PBS), the sections were incubated with anti-γH2AX primary antibody (1:1000; Beyotime Biotechnology, Shanghai, China) at 4 °C overnight. Subsequent immunohistochemical procedures were performed according to the manufacturer’s instructions, followed by hematoxylin counterstaining (Ligen, China). Stained sections were visualized and photographed using a Leica DM6 B microscope (Leica Microsystems, Wetzlar, Germany).

### 4.10. H&E Staining

Tumors were collected from mice bearing SW1990 xenografts. The samples were fixed in 4% paraformaldehyde, processed for paraffin embedding, and sectioned into 4-μm-thick slices. Hematoxylin and eosin (H&E) staining was performed using a commercial staining protocol provided by the manufacturer (Beyotime Biotechnology, Shanghai, China). The stained sections were examined using a Leica DM6B microscope (Leica Microsystems, Wetzlar, Germany) to assess histological changes in tumors and potential systemic toxicity caused by treatment. Representative microscopic images were obtained for analysis.

### 4.11. qRT-PCR Analysis

Total RNA was extracted from SW1990 cells and xenograft tumor tissues using TRIzol reagent (Vazyme Biotech Co., Ltd., Nanjing, China) according to the manufacturer’s protocol. Subsequently, 1.0 μg of purified RNA was reverse-transcribed into cDNA in a 20 μL reaction system using the HiScript II One-Step RT-PCR Kit (P612-01, Vazyme Biotech Co., Ltd., Nanjing, China). Quantitative real-time PCR (qRT-PCR) analysis was performed using ChamQ SYBR qPCR Master Mix (Q311-02, Vazyme Biotech Co., Ltd., Nanjing, China) on a QuantStudio™ 3 Real-Time PCR System (Applied Biosystems, Thermo Fisher Scientific, Waltham, MA, USA). Each reaction contained 1.0 μL of cDNA template, and all samples were analyzed in technical triplicates. No-template controls (NTCs) were included to monitor potential contamination. GAPDH was used as the endogenous control for normalization. Relative gene expression was determined using the 2^−ΔΔCt^ method and presented as fold changes compared to the control group. Primer sequences are provided in Table 3.

### 4.12. Gene Expression and Correlation Analysis

The expression profiles of PARP7, PARP1, and PARP2 in pancreatic cancer tissues and matched normal tissues were compared using data from The Cancer Genome Atlas (TCGA) database through Gene Expression Profiling Interactive Analysis version 2 (GEPIA2; http://gepia2.cancer-pku.cn accessed on 7 August 2026). Additionally, GEPIA2 was utilized to assess the correlations among gene expression levels in pancreatic adenocarcinoma samples. The relationship between gene expression patterns and overall survival of patients with pancreatic cancer was evaluated using Kaplan–Meier survival analysis based on TCGA data. Furthermore, functional enrichment analysis of key genes was conducted using the Gene Ontology (GO) database via the Omicsmart online platform to explore their biological and functional characteristics.

### 4.13. RNA Sequencing

SW1990 cells were treated with Olaparib (5.0 μM), XYL-1 (2.5 μM), or a combination of XYL-1 (2.5 μM) and Olaparib (5.0 μM) for 7 days. Following treatment, cells were harvested, and total RNA was extracted using TRIzol reagent (Vazyme, Nanjing, China) according to the manufacturer’s instructions. RNA integrity and purity were assessed using an Agilent 2100 Bioanalyzer (Agilent Technologies, Inc., Santa Clara, CA, USA); only RNA samples with a RIN ≥ 7.0, OD260/280 values between 1.8 and 2.1, and OD260/230 values greater than 1.8 were used for subsequent library construction. Briefly, 500 ng of qualified total RNA per sample was subjected to mRNA enrichment using oligo(dT) magnetic beads, followed by RNA fragmentation. RNA libraries were constructed using the NEBNext Ultra RNA Library Prep Kit for Illumina (New England Biolabs, Inc., Ipswich, MA, USA). Library quality was verified, and paired-end 150 bp sequencing was performed on an Illumina HiSeq 4000 platform by Gene Denovo Biotechnology Co., Ltd. (Guangzhou, China), yielding at least 6 G of clean reads per sample. Raw sequencing reads were filtered to remove low-quality reads, and clean reads were aligned to the human reference genome GRCh38 (GENCODE v38 annotation). Differentially expressed genes (DEGs) were identified using the thresholds of |log2(fold change)| > 1.0 and adjusted *p*-value < 0.05. Genes with log2FC > 1.0 were classified as upregulated, while those with log2FC < −1.0 were classified as downregulated.

### 4.14. Statistical Analysis

All experiments were independently repeated at least three times, and results are expressed as mean ± standard deviation (SD). Statistical analyses were performed using GraphPad Prism 8.0 software (GraphPad Software Inc.). Comparisons between two groups were conducted using an unpaired Student’s *t*-test or one-way ANOVA. Statistical significance was defined as a *p* value < 0.05.

## Figures and Tables

**Table 1:** CI values for the effects of combining XYL-1 with Olaparib on the proliferation of SW1990 cells after 7 days.

Cell			SW1990 Ratio (1:1)		SW1990 Ratio (1:2)		SW1990 Ratio (1:4)	
Group	XYL-1	Olaparib	XYL-1	Olaparib	XYL-1	Olaparib	XYL-1	Olaparib
IC_50_ (μM)	45.3 ± 5.2	12.5 ± 0.4	3.2 ± 0.5 **	3.2 ± 0.5 ^#^	1.2 ± 0.3 **	2.4 ± 0.4 ^##^	2.1 ± 0.6 **	8.4 ± 0.8 ^#^
CI value	/	/	0.33 ± 0.06		0.22 ± 0.03		0.72 ± 0.45	

Values are mean ± SD (*n* = 3 for each replicate) of three independent experiments. ** *p* < 0.01 compared to the XYL-1 alone treated group, ^#^ *p* < 0.05 and ^##^ *p* < 0.01 compared to the Olaparib alone treated group.

**Table 2:** CI values for the effects of combining XYL-1 with Olaparib on the proliferation of CFPAC cells after 7 days.

Cell			CFPAC Ratio (1:1)		CFPAC Ratio (1:2)		CFPAC Ratio (1:4)	
Group	XYL-1	Olaparib	XYL-1	Olaparib	XYL-1	Olaparib	XYL-1	Olaparib
IC_50_ (μM)	52.3 ± 3.7	9.3 ± 0.7	4.2 ± 0.5 **	4.2 ± 0.5 ^#^	1.2 ± 0.4 **	2.3 ± 0.6 ^##^	1.7 ± 0.2 **	6.8 ± 0.3 ^#^
CI value	/	/	0.53 ± 0.16		0.27 ± 0.06		0.76 ± 0.23	

Values are mean ± SD (*n* = 3 for each replicate) of three independent experiments. ** *p* < 0.01 compared to the XYL-1 alone treated group, ^#^ *p* < 0.05 and ^##^ *p* < 0.01 compared to the Olaparib alone treated group.

**Table 3:** Primer sequences for genes in qRT-PCR.

Name	Sense (5′-3′)	Antisense (5′-3′)
SCD1	CTTGCGATATGCTGTGGTGC	CCGGGGGCTAATGTTCTTGT
BRCA1	TACTAGGCATAGCACCGTTG	ATGCCTTTGCCAATATTACCTG
GAPDH	GAAACTGTGGCGTGATGGC	CACCACTGACACGTTGGCAG

## Data Availability

Data are available on reasonable request.

## Supplementary Materials

The following supporting information can be downloaded at: https://www.mdpi.com/article/10.3390/molecules31162781/s1, Table S1: Inhibitory activity on PARP7 of XYL-1. Table S2: CI values for the effects of combining I-1 with Olaparib on the proliferation of SW1990 cells after 7 days. Table S3: CI values for the effects of combining XYL-1 with Olaparib on the proliferation of HPDE6-C7 cells after 7 days. Figure S1: The effects of XYL-1 and/or Olaparib on RAD51 foci formation. Representative immunofluorescence images showing RAD51 foci. Figure S2: Effect of knockdown of SCD1 on the proliferation of SW1990 cells. Figure S3: The effects of SCD1 knockdown on RAD51 foci formation in SW1990 cells. Representative immunofluorescence images showing RAD51 foci. Figure S4: Effect of BRCA1 overexpression on the proliferation of SW1990 cells. Data are presented as mean + SD, *n* = 5 mice per group from a single experiment. Statistical significance was deter-mined by one-way analysis of variance (one-way ANOVA). ** *p* < 0.01 compared with control group. Figure S5: The effects of the overexpression of BRCA1 on RAD51 foci formation in SCD1-knockout SW1990 cells. Representative immunofluorescence images showing RAD51 foci. Data are presented as mean + SD, *n* = 5 mice per group from a single experiment. Statistical significance was deter-mined by one-way analysis of variance (one-way ANOVA). ** *p* < 0.01 compared with control group. Figure S6: Effects of I-1 and/or Olaparib on the expression of SCD1 and BRCA1 in the SW1990 cells model. (A) The expression of SCD1 mRNA in the SW1990 cells model treated with I-1 and/or Olaparib was detected using qRT-PCR. (B) The expression of BRCA1 mRNA in the SW1990 cells model treated with XYL-1 and/or Olaparib was detected using qRT-PCR. (C) The expression levels of SCD1 and BRCA1 proteins in the SW1990 cells model treated with XYL-1 and/or Olaparib were detected using Western blotting. (D) The gray values of the SCD1 protein bands were quantitatively analyzed using ImageJ software, normalized to β-tubulin as a reference. (E) The gray values of the BRCA1 protein bands were quantitatively analyzed using ImageJ software, normalized to β-tubulin as a reference. Data are presented as mean + SD, *n* = 5 mice per group from a single experiment. Statistical significance was deter-mined by one-way analysis of variance (one-way ANOVA). * *p* < 0.05 and ** *p* < 0.01 compared with control group, ## *p* < 0.01 compared with the combination group of XYL-1 and Olaparib.

## Abbreviations

The following abbreviations are used in this manuscript:

PAAD	Pancreatic adenocarcinoma
HR	Homologous recombination
PARP7	Diphtheria toxin-like ART14
DDR	DNA damage response
SSBs	Single-strand breaks
DSBs	Double-strand breaks
BER	Base excision repair
PARP1/2	Poly (ADP-ribose) polymerase 1/2
