# Supplementary material for: XYL-1 and Olaparib Synergistically Inhibit the Growth of Pancreatic Cancer by Suppressing the SCD1/BRCA1 Signaling Pathway
Source: Molecules. 2026 Aug 10;31(16):2781. doi: 10.3390/molecules31162781 (PMC13515869; doi:10.3390/molecules31162781)
Supplement: Supplementary file 1 [file molecules-31-02781-s001.zip › molecules-4453040-supplementary.pdf]

Supplementary Information

XYL-1 and Olaparib Synergistically Inhibit the Growth of Pancreatic Cancer by Suppressing the SCD1/BRCA1 Signaling Pathway

Ye Yang[1], Lei Huang[1*], Yaru Du[2], Qingyue Zhu[2], Li Dai[3], Bingjun Qian[1, 3*]

[1] Department of Pharmacology and Medicinal Chemistry, Jiangsu Medical College, Yancheng 224005, China;

[2] Department of Chinese Traditional Medicine, Nanjing University of Chinese Medicine, Nanjing 210023, China

[3] Department of Medicinal Chemistry, Jiangsu Medical College, Yancheng 224005, China;

* Correspondence: bjqianfd@jsmc.edu.cn (B.Q.); 3120010012@stu.cpu.edu.cn (L.H.)

Table S1. Inhibitory activity on PARP7 of XYL-1

Compd	Inhibitory rate @100 nM[a] (PARP7)	PARP7 (IC_{50}): nM
XYL-1	102.5 ± 0.8%	0.61 ± 0.45
RBN-2397	—	6.30± 0.27

Table S2. CI values for the effects of combining I-1 with Olaparib on the proliferation of SW1990 cells after 7 days

**Cell**			**SW1990 Ratio (1:1)**		**SW1990 Ratio (1:2)**		**SW1990 Ratio (1:4)**	
Group	I-1	Olaparib	I-1	Olaparib	I-1	Olaparib	I-1	Olaparib
IC_{50} (μM)	50.3±6.8	15.6±0.3	2.5±0.6**	2.5±0.6$^{\#}$	0.9±0.4**	1.8±0.2$^{\#\#}$	1.7±0.3**	6.8±0.5$^{\#}$
CI value	/	/	0.21±0.03		0.13±0.04		0.47±0.02	

*P<0.05 and **P<0.01 compared to XYL-1 alone treated group, $^{\#}$P<0.05 and $^{\#\#}$P<0.01 compared to Olaparib alone treated group

Table S3. CI values for the effects of combining XYL-1 with Olaparib on the proliferation of HPDE6-C7 cells after 7 days

Cell	HPDE6-C7	HPDE6-C7	HPDE6-C7

			Ratio (1:1)		Ratio (1:2)		Ratio (1:4)	
Group	XYL-1 (μM)	Olaparib (μM)	XYL-1 (μM)	Olaparib (μM)	XYL-1 (μM)	Olaparib (μM)	XYL-1 (μM)	Olaparib (μM)
Mean±SD	78.5±2.4	63.2±3.5	43.6±3.2	43.6±3.2	36.7±5.4	73.4±2.6	26.7±6.2	106.8±8.3#
CI value	/	/	1.2±0.3		1.6±0.4		2.0±0.2	

**P<0.01 compared to XYL-1 group, #P<0.05 and ##P<0.01 compared to Olaparib group

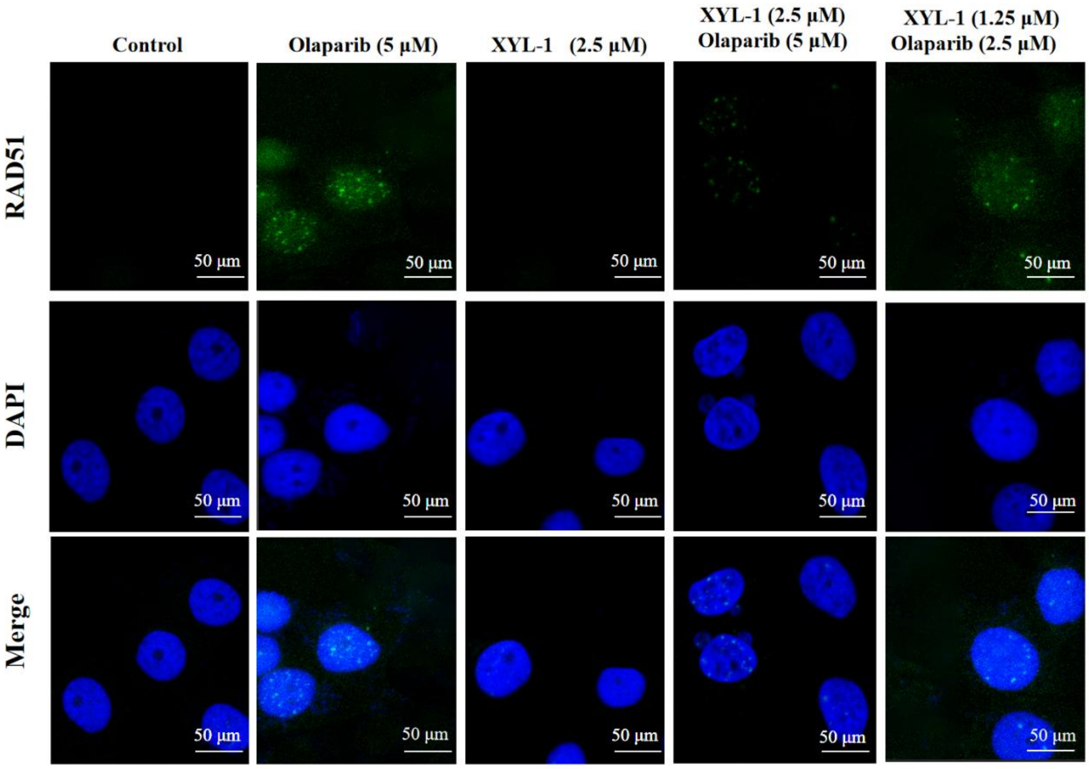

Figure S1. The effects of XYL-1 and/or Olaparib on RAD51 foci formation. Representative immunofluorescence images showing RAD51 foci.

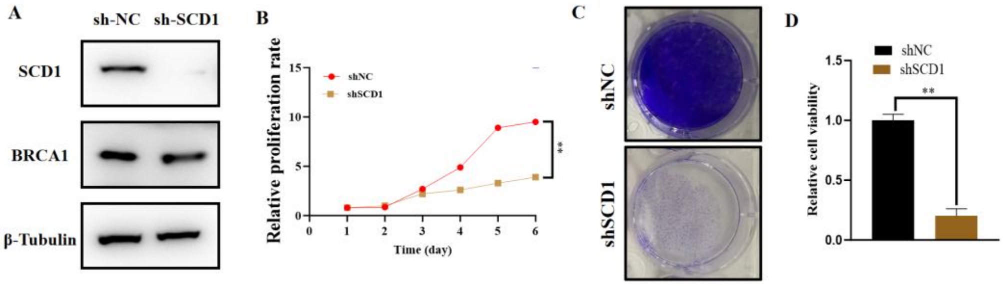

Figure S2. Effect of knockdown of SCD1 on the proliferation of SW1990 cells.

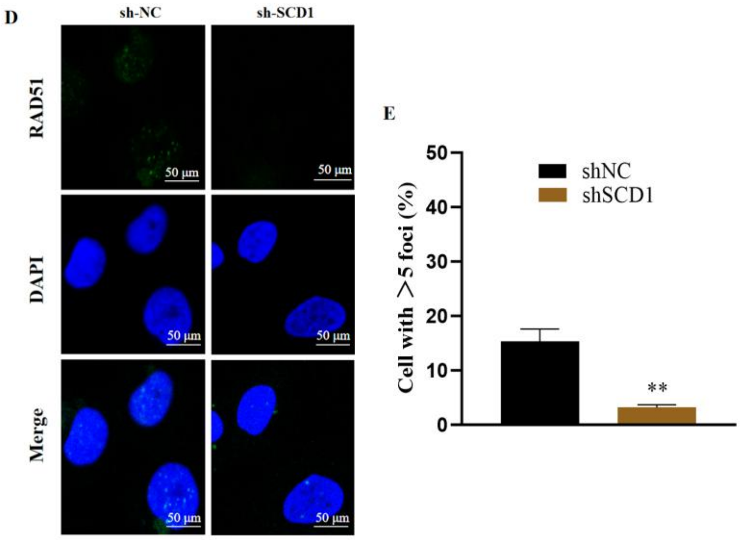

Figure S3. The effects of SCD1 knockdown on RAD51 foci formation in SW1990 cells. Representative immunofluorescence images showing RAD51 foci.

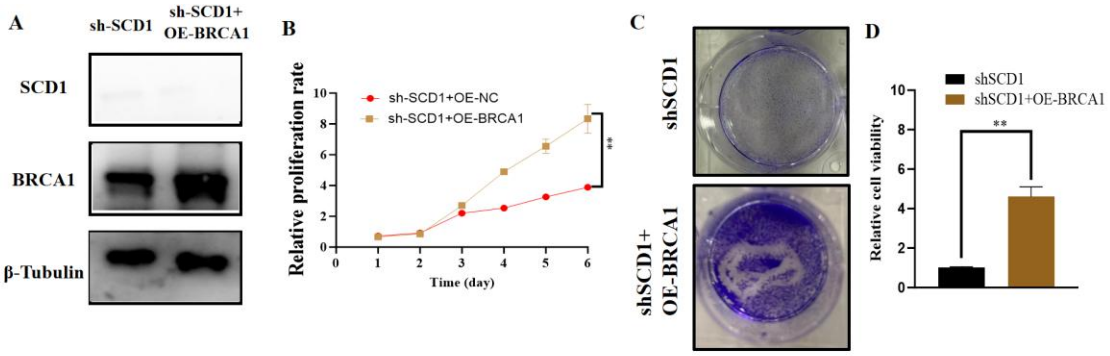

Figure S4. Effect of BRCA1 overexpression on the proliferation of SW1990 cells. Data are presented as mean + SD, n = 5 mice per group from a single experiment. Statistical significance was deter-mined by one-way analysis of variance (one-way ANOVA). ** $p < 0.01$ compared with control group.

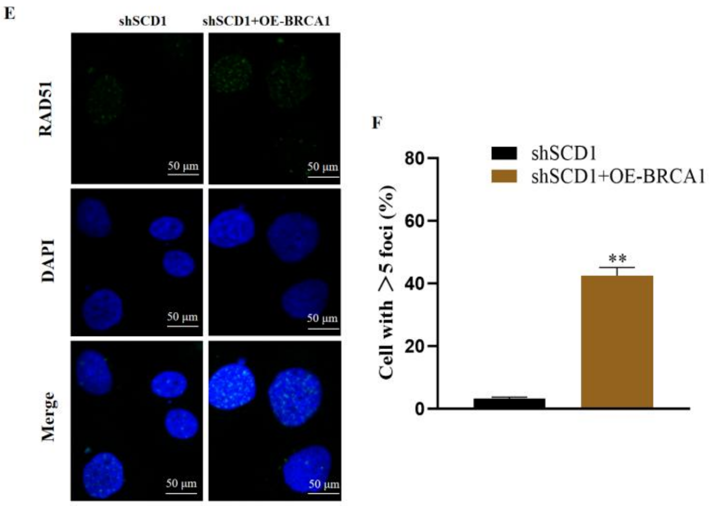

Figure S5. The effects of the overexpression of BRCA1 on RAD51 foci formation in SCD1-knockout SW1990 cells. Representative immunofluorescence images showing RAD51 foci. Data are presented as mean + SD, n = 5 mice per group from a single experiment. Statistical significance was deter-mined by one-way analysis of variance (one-way ANOVA). ** $p < 0.01$ compared with control group.

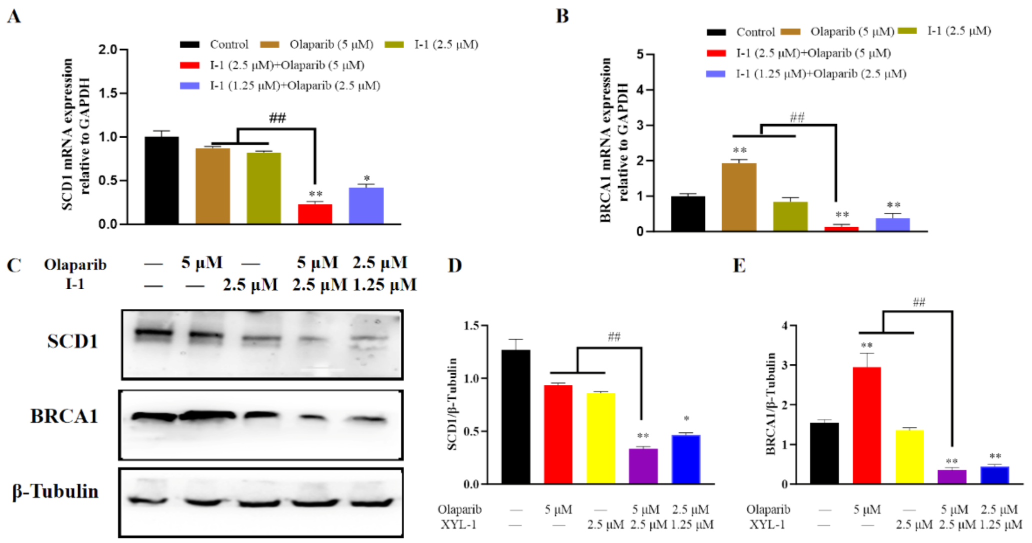

Figure S6. Effects of I-1 and/or Olaparib on the expression of SCD1 and BRCA1 in the SW1990 cells model. (A) The expression of SCD1 mRNA in the SW1990 cells model treated with I-1 and/or Olaparib was detected using qRT-PCR. (B) The expression of BRCA1 mRNA in the SW1990 cells model treated with XYL-1 and/or Olaparib was detected using qRT-PCR. (C) The expression levels of SCD1 and BRCA1 proteins in the SW1990 cells model treated with XYL-1 and/or Olaparib were detected using Western blotting. (D) The gray values of the SCD1 protein bands were quantitatively analyzed using ImageJ software, normalized to β-tubulin as a reference. (E) The gray values of the BRCA1 protein bands were quantitatively analyzed using ImageJ software, normalized to β-tubulin as a reference. Data are presented as mean + SD, n = 5 mice per group from a single experiment. Statistical significance was deter-mined by one-way analysis of variance (one-way ANOVA). * $p < 0.05$ and ** $p < 0.01$ compared with control group, $^{\#\#}$ $p < 0.01$ compared with the combination group of XYL-1 and Olaparib.
